# Pleura-pneumonia Contagiosa

**Published:** 1891-05

**Authors:** 


### CONTAGIOUS PLEURO-PNEUMONIA.

### COST OF SUPPRESSION IN ILLINOIS.

The following letter from the Secretary of the State Board of Live Stock Commissioners of Illinois, is of interest as a record of the number of animals which were quarantined and slaughtered in the outbreak of the lung plague in Illinois, together with the cost of inspection and indemnity. It will be of interest to hear more upon the subject and to learn what return in value of usable parts of the beeves was made, and what disposition of the carcasses was required.

State of Illinois, State Board of Live Stock Commissioners.

Springfield, April 14, 1891.

John W. Gadsden, M. R. C. V. S.

128 N. 10th St., Philadelphia, Pa.

My Dear Sir :—

Your letter of the 10th inquiring as to the cost of stamping out the outbreak of Contagious Pleuro-pneumonia in Cook County, 1886-88, is received, and I have endeavored to compile the figures as accurately as possible, within the brief time, without going into the expense of vouchers for the detail expenditures in order that the expense items might be exact. There were slaughtered, diseased and exposed cattle, by order of this Board, or by the owners on permits issued by the Board, and inspected by our veterinarians, 10,732 cattle; 107 died from the disease between the period of quarantine and the date of the commencement of slaughter, making the grand total, 10,839. The State of Illinois paid $35,730.75 for 3,032 head of these cattle, an average of $11,78 per head, while the United States Government paid $19,737.31 for 1,177 head, or an average of $17.66 per head. So that, of the 10,839 head, 4,209 head were paid for; 107 head died from the disease, 6,523 head were slaughtered by owners on permit, the State paying for 1,855 head more than were paid for by the Government, and the average amount paid by the State per head was $5.88 less than the amount per head paid by the Government. The expenses of the suppression of this outbreak other than for damages for animals slaughtered, paid by the State, in round numbers, was about $20,071, while the amount of expenses other than for damages for slaughtered animals paid by the Government was $60,106.21, as appears from the reports of the Bureau for 1887-88. The total amount for all purposes, in the suppression of the outbreak, paid by the State, was about $55,801.75, and by the Government $79,843.52, or a grand total of $135,645.27.

This outbreak was discovered September, 12, 1886, and the last case of disease found was December 29, 1887, a period of one year, three months and seventeen days, and quarantine restrictions were removed three months and two days later, or on April 1, 1888.

While the Chief of the Bureau of Animal Industry, in endeavoring to explain the reasons, in the report of 1887, for the very great difference in the amounts paid by the Government for slaughtered animals, and for other expenses of suppressing the outbreak, gives some legitimate reasons for this, one of the principal reasons, in our opinion, is omitted, and is to be found fully set forth in the report of this Board for 1887, wherein a sample of the work done by the employes of the Bureau of Animal Industry is described; it having required twenty deputy sheriffs, and three veterinarians, in the employ of the Bureau, forty-eight days to quarantine 1,217 head of cattle in 357 stables at a cost of $3,300, or $2.75 per head, while, shortly thereafter, the employes of this Board, in the same locality, with but fourteen men, in thirty-five days, quarantined and registered 10,192 cattle in over 7,000 stables at a cost of but ten cents per head. Very much, also, of this expense, endeavored to be satisfactorily explained by the Chief of the Bureau, is due to the policy inaugurated and carried out by him during the last six or seven months of the "War," contrary to the advice and wishes of this Board, and his chief inspector at Chicago, Prof. James Law, of stopping the enforced slaughter of exposed animals, and permitting the disease, in the language of the Chief Inspector, "to burn itself out," while a large corps of veterinary inspectors were kept inspecting and re-inspecting the exposed animals in quarantine, all of which eventually had to be killed, after a useless delay of many months.

Trusting the above information is what you desire, and that it will prove advantagious to your purpose, I remain,

Very respectfully yours,

B. Johnson, *Seeretary*

